# Case Report/Case Series: Rare case of anti-LGI1 limbic encephalitis with rapidly progressive dementia, psychiatric symptoms, and frequently seizures: A case report

**DOI:** 10.1097/MD.0000000000026654

**Published:** 2021-07-23

**Authors:** Haiyan Wu, Fan Mei, Lixin Liu, Li Zhang, Hongjun Hao, Shouzi Zhang

**Affiliations:** a Psychiatry Department, Beijing Geriatric Hospital, Beijing, PR China; b Institute of Systems Biomedicine, Peking University Health Science Center, Beijing, PR China; c Department of Neurology, Peking University, First Hospital, Beijing, PR China.

**Keywords:** anti-leucine-rich glioma-inactivated 1 limbic encephalitis (anti-LGI1 LE), autoimmune encephalitis, faciobrachial dystonic seizures, rapidly progressive dementia

## Abstract

**Rationale:**

Anti leucine-rich glioma inactivated 1 (LGI1) limbic encephalitis (LE) is rare autoimmune encephalitis, characterized by acute or subacute cognitive impairment, faciobrachial dystonic seizures, mental disorders, and refractory hyponatremia. As a type of treatable rapidly progressive dementia with a good prognosis, early, and accurate diagnosis is essential. We present a case of anti-LGI1 LE who was initially misdiagnosed with Alzheimer disease because his clinical manifestations were similar to Alzheimer disease.

**Patient concerns:**

A male patient presenting with rapidly progressive dementia, faciobrachial dystonic seizures, psychiatric disturbance, and refractory hyponatremia was admitted. The scores of Mini-Mental State Examination, Montreal Cognitive Assessment, and Neuropsychiatric Inventory were 19/30, 16/30, and 91/144, respectively. Brain magnetic resonance images indicated moderate atrophy of the hippocampus and abnormally hyperintensities in the left medial temporal and hippocampus.

**Diagnosis:**

The patient was diagnosed with anti-LGI1 LE based on the presence of LGI-1 antibodies in the cerebrospinal fluid and serum and clinical manifestations.

**Interventions:**

Patient was treated with glucocorticoid against LGI1, antiepileptic drug, cholinesterase inhibitors, and other adjuvant therapy.

**Outcomes:**

The patient showed marked improvement on immunotherapy. Clinical symptoms were disappeared and the LGI-1 antibodies in cerebrospinal fluid and serum were both negative at the time of discharge.

**Conclusions:**

Recognition of the specific symptoms and LGI-1 antibody test will be helpful for the early diagnosis, prompt immunotherapy, and good prognosis. This case raises the awareness that rapidly progressive dementia with frequent seizures could be caused by immunoreactions.

## 1 Introduction

Autoimmune encephalitis (AE) is a new type of neurological autoimmune disease directed by the autoantibodies of the neuronal cell surface or intracellular antigens. Different subtypes of AE are distinguished by specific autoantibodies.^[1]^ Anti-leucine-rich glioma-inactivated 1 limbic encephalitis (anti-LGI1 LE) is a rare and treatable AE discovered in recent years, which is caused by the involvement of LGI1 antibody.^[2,3]^ The distinctive clinical features of anti-LGI1 LE are rapidly progressive dementia, faciobrachial dystonic seizures (FBDS), refractory hyponatremia, and mental disorders.^[4–7]^ It is often misdiagnosed as Alzheimer disease or other types of dementia in the early stage, in that patients will not be treated with immunotherapy promptly.^[8]^ We here present an anti-LGI1 LE case that exhibited prominent rapidly progressive dementia, psychiatric disturbances, FBDS and serum, and cerebrospinal fluid (CSF) testing positive for anti-LGI1 antibodies.

## 2 Case presentation

A 69-year-old male was present to the Department of Psychiatry with a 4-month history of cognitive impairments and psychiatric disturbances. The patient exhibited recent rapid memory decline, language function impairment, disorientation of time and place, and executive dysfunction. He also presented behavioral psychiatric symptoms include delusions, hallucinations, obvious anxiety and depression, agitation, and irritability. His sleep disorder was apparent, with occasionally yelling and dancing limbs, nightmares, and bedwetting behavior several times. A brain magnetic resonance imaging showed bilateral frontal and parietal cortex atrophy and hippocampal atrophy. Patient was initially diagnosed with Alzheimer disease, anxiety, depression, and sleep disorder, and treated with memantine hydrochloride and duloxetine hydrochloride enteric. However, symptoms were not improved. He has been suffered from right upper limb convulsive seizures without unconscious for 20 to 30 times per day and every seizure lasted about 1 to 2 seconds.

After several falls and serious seizures, the patient was admitted to our psychiatry ward. The main clinical characteristics of the patient were FBDS, rapidly progressive cognitive impairment, and behavioral psychiatric disorders. On admission, the scores of Mini-Mental State Examination, Montreal Cognitive Assessment, and Neuropsychiatric Inventory were 19/30, 16/30, and 91/144, respectively, suggesting that the patient had moderate cognitive impairment and serious mental disorders. The neurological examination was unremarkable. FBDS occurred up to 30 to 40 times a day, and the antiepileptic therapy failed to control the seizures. At the same time, the patient was suffered from refractory hyponatremia and he was treated with intravenous normal saline and oral sodium tablets.

The patient was a retired engineer with a university degree. He had a history of hypertension for 10 years, no history of autoimmune diseases, such as thromboembolic vasculitis. There was no family history of dementia or other neurologic diseases.

**Figure 1:**
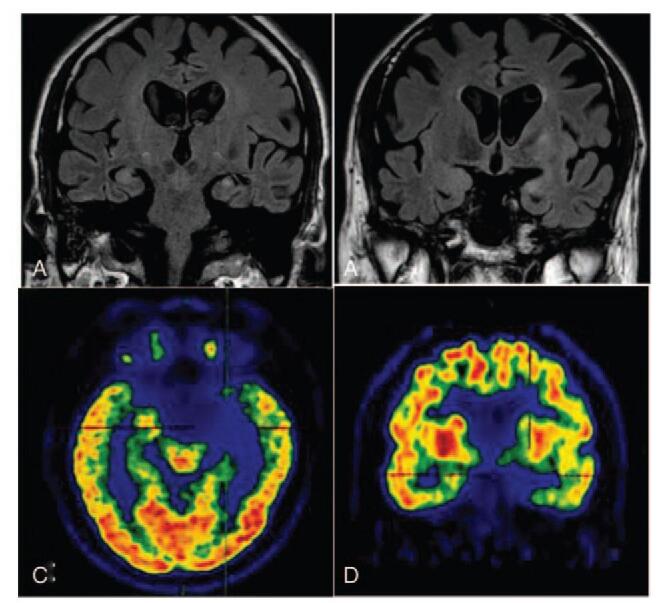
Patient's neuroimaging. (A) T2W FLAIR showed the abnormal hyperintensities in the left medial temporal and hippocampus. (B) T2W FLAIR showed moderate atrophy of the hippocampus with MTA level 3. (C) PET/CT showed the partial absence of radioactivity in the left medial temporal cortex and hippocampus. (D) PET/CT showed the radioactivity is obviously less than the opposite side in the left caudate nucleus head. CT = computed tomography, MTA = medial temporal lobe atrophy, PET = positron emission tomography, T2W FLAIR = T2-weighted fluid-attenuated inversion recovery.

**Figure 2:**
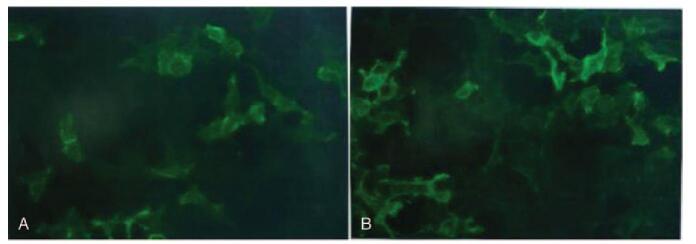
Results of LGI1 antibody in CSF and serum. (A) CSF: VGKC/LGI1-1/transfected cells: positive +++. (B) Serum: VGKC/LGI1-1/transfected cell: positive +++ (1:10 titer dilution). CSF = cerebrospinal fluid, LGI1 = leucine-rich glioma inactivated 1, VGKC = voltage-gated potassium channels.

The patient's brain magnetic resonance images indicated abnormally hyperintensities in the left medial temporal and hippocampus (Fig. 1A). The medial temporal lobe atrophy score was 3, indicating the moderate atrophy of hippocampus (Fig. 1B). His fluorine-18-fluorodeoxyglucose positron emission tomography showed the partial absence of radioactivity in the left medial temporal cortex and hippocampus (Fig. 1C). The left caudate nucleus head was less radioactive than the opposite side (Fig. 1D). No thymoma and other tumors were found by chest computed tomography and the whole body positron emission tomography examination. The routine biochemical tests of CSF were negative. However, the LGI-1 antibodies in both CSF and serum were strongly positive by cell-based transfection immunofluorescence assay. This satisfies the diagnostic criteria of anti-LGI1 LE (Fig. 2). The other immune indexes, such as glutamic acid receptor antibodies (NMDA/transfection cells, AMPA1/transfection cell, and AMPA2/transfection cell), voltage-gated potassium channels/contact protein-related protein 2 antibodies/transfection cells, were all negative.

**Figure 3:**
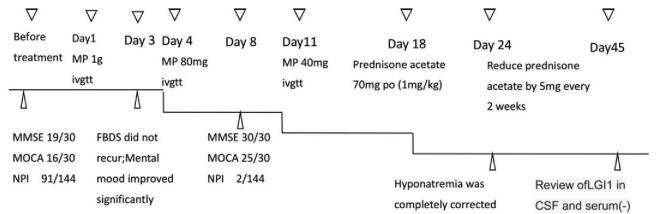
Treatment and efficacy of the patients.

Therefore, the patient was diagnosed with anti- LGI1 LE and the diagnosis of active tuberculosis and tumor were excluded. Methylprednisolone therapy was intravenously administrated for 17 days (methylprednisolone 1 g for 3 days, 80 mg for 7 days, and 40 mg for 7 days), followed by a sequential oral prednisone acetate for 6 months (begin with 1 mg/kg/d, 70 mg/d, reducing by 5 mg every 2 weeks). In addition, the antiepileptic medication (Carbamazepine), correction of hyponatremia, and other supportive treatments were administrated. The psychological counseling, entertainment treatment, cognitive training, horticultural therapy, and other non-drug treatments were administered simultaneously. After 3 days of IV methylprednisolone treatment, the FBDS completely disappeared. On the 8th day of the treatment, the patients’ cognitive function almost returned to normal, with Mini-Mental State Examination score 30/30 and Montreal Cognitive Assessment score 25/30. Moreover, psychiatric symptoms were significantly improved with Neuropsychiatric Inventory score 2/144. Hyponatremia was corrected after 24 days. On the 45th day of hormone therapy, the tests for LGI1 antibodies in CSF and serum were negative (Fig. 3).

## 3 Discussion and conclusions

We report a case of anti-LGI1 LE that presented rapidly progressive dementia, psychiatric symptoms, and FBDS. Patient was initially diagnosed with cognitive impairment and mental disorder by other hospitals and treated with a variety of antipsychotic drugs, however, the symptoms were persistent. He came to our hospital for further investigations and consultation. During his hospitalization, FBDS symptoms and refractory hyponatremia were noted. We noticed that patient's diet was normal and he had no history of metabolic and endocrine diseases, however, the hyponatremia could not be corrected. In view of his clinical presentations of rapid progressive cognitive decline, FBDS symptoms, and refractory hyponatremia, serological markers for autoimmune encephalitis were requested. The patient has later confirmed diagnosis of anti-LGI1 LE based on the positive anti-LGI1 antibodies in the CSF and serum. Appropriate treatment was initiated and the prognosis was excellent. In addition, considering the older age of the patients, we implemented individualized treatment. Different from previous studies that used 6-day hormone shock therapy, we reduced the dose to routine hormone infusion after 3 days of hormone shock therapy, combined with a variety of non-drug therapy regimens. At present, the patient has been followed up for 2 years, and the prognosis was good.

As a type of rapidly progressive dementia (RPD), LE was usually misdiagnosed. Prion diseases are the prototypical causes of RPDs. Aside from prion diseases, the other common causes of RPD are atypical presentations of some neurodegenerative disorders such as AD, frontotemporal dementia or vascular dementia, some infections, and neoplasms. As the neurologic autoimmune tests became more applicable, it is likely that antibody-mediated encephalopathies are an even larger percentage of non-prion RPDs than ever shown. Among treatable RPDs cases identified in a cohorts study,^[9]^ 37% of RPDs were immune-mediated, 35% were neoplasms, 20% were infections, and 8% were metabolic disorders.

LG1 is the target antigen for LGI1 antibody-mediated limbic encephalitis.^[13–15]^ LGI 1 is a secretory synaptic glycoprotein mainly distributed in the temporal cortex and cornu ammonis 3,^[10]^ a hippocampal subfield that has been implicated in memory encoding.^[11,12]^ A recent research^[13]^ suggests that significant bilateral atrophy of the hippocampus and its subfields, as well as significantly impaired hippocampal microstructural integrity, was observed in anti-LGI1 LE. The antigen-antibody reaction in anti-LGI1 LE leads to inflammatory changes in the hippocampus, which eventually results in atrophy or sclerosis of the hippocampus and the rapid decline of cognitive function. In the early stage of this disease, it is often misdiagnosed with other types of dementia or psychiatric disturbances, and the treatment of anti-dementia and antipsychotic drugs is used, this will delay the accurate diagnosis and immunotherapy of the disease. It results in a poor prognosis for the patients.^[4,16,17]^ The structural damage may be correlated with persistent verbal and visuospatial memory deficits. Patients with delayed treatment had worse verbal and visuospatial memory performance, while early immunotherapy was associated with better memory outcomes.^[13]^ It is very important to distinguish LE from other untreatable RPDs such Creuzfeldt–Jakob disease. In our case, the patient had moderate atrophy of the hippocampus, decreased function in multiple cognitive domains, and abnormal mental behaviors. After hormone immunotherapy, his clinical symptoms in cognitive and mental aspects improved significantly.

In addition to cognitive decline, the seizure is another major clinical feature of anti-LGI1 LE, with an incidence of 65% to 82%.^[3,18]^ FBDS are the characteristic seizure type that presenting as short, stereotyped dystonic movements of the face and the ipsilateral arm and/or leg, frequently precede the onset of anti-LGI1 encephalitis.^[19,20]^ Because LGI1 is a part of voltage-gated potassium channel complex which can interact with presynaptic and postsynaptic related proteins and form across synapses protein complexes,^[18]^ LGI1 antibody can inhibit ligand-receptor interactions between LGI1 and presynaptic or postsynaptic proteins by acting on the epitope and leucine repeat domain of LGI1. It leads to decrease inhibitory neurons and increase neural excitability and results in epileptic seizures.^[21,22]^ Duration of FBDS was associated with reduced pallidum volume.^[13]^ On clinical evaluation, 20% of the patients presented with pilomotor or autonomic seizures. These subtle clinical symptoms should be considered as possible early signs of limbic encephalitis. Early recognition and immunosuppressive treatment of FBDS can prevent progression to limbic encephalitis and the development of cognitive deficits.^[23–25]^ Due to its subtle clinical symptoms, it is often miss diagnosed at the early stage of the disease. Our case had typical FBDS characteristics but did not attract the attention of the patient himself and his family members before hospitalization. During hospitalization, we noticed the symptoms of FBDS and traced the history further, and confirmed that the FBDS symptoms existed in the early course of his illness. The patient had a remarkable response to immunotherapy. FBDS was completely controlled after 3 days of the treatment with methylprednisolone.

Early diagnosis and prompt treatment are crucial to avoid long-term sequelae, including psycho-cognitive deficits and persisting seizures. LE with paucisymptomatic electro-clinical presentation seemed to be more associated with chronic epilepsy than LE presenting with definite and severe “limbic syndrome.”^[26]^

In summary, we show that anti-LGI1 LE is reversible and treatable. As treatment of rapidly progressive dementia is entirely dependent on the diagnosis, a comprehensive, structured, but pragmatic approach to diagnosis, including key clinical, laboratory, and radiologic features is critical. For better outcomes and patient's quality of life. The patient had a good response to immunotherapy, and individualized therapy and non-pharmacologic therapy should also be given. FBDS normally improved faster than cognitive dysfunction and hyponatremia. After the clinical symptoms disappear and the laboratory index is normal, the review of LGI antibody can be used for the indicators to evaluate the treatment effect. Furthermore, we should follow up with the patient for a long time to monitor the possibility of recurrence.

## Acknowledgments

We are very grateful to all the participants of the study. We thank Dr HH for the technical assistance.

## Author contributions

SZ designed the study strategy. LL, LZ, and HW performed the assessment of the data collection. HH completed immunological testing works. HW and FM wrote the manuscript. All authors reviewed the manuscript.

**Funding acquisition:** Shouzi Zhang.

**Project administration:** Li Zhang, Hongjun Hao.

**Resources:** Lixin Liu.

**Writing – original draft:** Haiyan Wu.

**Writing – review & editing:** Haiyan Wu, Fan Mei, Shouzi Zhang.
